# Chronic draining xiphisternal fistula associated with fractured right coronary artery stent and transdiaphragmatic inflammatory extension: a multimodality imaging and surgical correlation

**DOI:** 10.1186/s43044-026-00770-3

**Published:** 2026-07-24

**Authors:** Zakiur Rehman Ansari, Zainul Abedein Hamdulay, Azizullah Khan, Sanjesh Jain, Meher Hamdulay

**Affiliations:** 1 Department of Clinical Research in Cardiovascular Surgery and Critical Care Medicine, Masina Heart Institute, Mumbai, Maharashtra India; 2 Department of Cardiothoracic Surgery, Masina Heart Institute, Mumbai, India; 3 Department of Critical Care Medicine, Masina Heart Institute, Mumbai, India; 4 Department of Cardiac Anaesthesia, Masina Heart Institute, Mumbai, India

**Keywords:** RCA stent fracture, Coronary stent-related inflammatory process, Mediastinal inflammatory fistula, Chest wall sinus, CT coronary angiography, Cardiac MRI, Multimodality imaging

## Abstract

**Background:**

Delayed coronary stent-related inflammatory complications following percutaneous coronary intervention (PCI) are exceptionally rare, and chronic cutaneous fistulization years after stent implantation has only rarely been described. Such indolent presentations may mimic superficial chest wall infections, making diagnosis challenging. Multimodality imaging is essential for accurate diagnosis and surgical planning.

**Case presentation:**

A 53-year-old man with type 2 diabetes mellitus and ischemic heart disease underwent right coronary artery (RCA) stenting in 2018. He presented with recurrent intermittent fever and a persistent draining xiphisternal sinus for seven months following incision and drainage of a presumed chest wall abscess. Ultrasonography demonstrated a localized subcutaneous collection. Contrast-enhanced computed tomography and CT coronary angiography (CTCA) revealed a fractured, chronically occluded RCA stent with extensive peri-stent inflammatory soft tissue extending into the pericardium, diaphragm, and subdiaphragmatic region. Cardiac magnetic resonance imaging confirmed transdiaphragmatic inflammatory extension and a fistulous tract communicating with the overlying skin. Transesophageal echocardiography was not performed because transthoracic echocardiography and cross-sectional imaging adequately delineated the lesion and showed no evidence of endocarditis. Following preoperative and perioperative antibiotic therapy, the patient underwent surgical excision of the fractured stent, chronic fibro-inflammatory tissue, and sinus tract. Intraoperative cultures and GeneXpert testing were negative, possibly reflecting prior prolonged antibiotic therapy. The postoperative course was uneventful, with complete wound healing, resolution of fever, and follow-up transthoracic echocardiography demonstrating no pericardial collection or valvular vegetations.

**Conclusions:**

This case highlights an exceptionally rare delayed culture-negative chronic peri-stent inflammatory fistulizing process associated with RCA stent fracture presenting nearly seven years after PCI. It emphasizes the importance of maintaining a high index of suspicion in patients with persistent chest wall sinuses after coronary intervention and demonstrates the complementary role of CTCA and cardiac magnetic resonance imaging in defining disease extent and guiding successful surgical management.

## Background

Coronary artery stent implantation is a cornerstone in the management of ischemic heart disease. Although percutaneous coronary intervention (PCI) is generally safe, coronary stent-related inflammatory or infective complications are exceptionally rare and are associated with substantial morbidity and mortality [1]. These complications may present with sepsis, coronary abscess formation, pseudoaneurysm, or acute coronary syndromes [2]. Contemporary scientific statements have further emphasized the importance of prompt recognition and multidisciplinary management of cardiovascular device-related infections [3].

Advances in multimodality imaging, particularly computed tomography coronary angiography (CTCA) and cardiac magnetic resonance imaging (MRI), have significantly improved the evaluation of complex peri-coronary inflammatory processes by enabling accurate assessment of stent integrity, soft-tissue involvement, disease extent, and fistulous tracts, thereby facilitating diagnosis and surgical planning [4, 5].

Most reported coronary stent-related inflammatory or infective complications occur within weeks of PCI and are attributed to procedural contamination, transient bacteremia, or immunocompromised states [1, 2]. In contrast, delayed presentations occurring several years after stent implantation are exceedingly uncommon and often diagnostically challenging because of their indolent clinical manifestations [6, 7].

Mechanical complications such as coronary stent fracture may contribute to chronic endothelial injury, altered local hemodynamics, persistent peri-stent inflammation, and adverse vascular remodeling [8, 9]. In rare circumstances, these chronic inflammatory processes may extend beyond the coronary artery into adjacent mediastinal structures and culminate in fistulous communication with surrounding tissues. However, chronic cutaneous xiphisternal fistulization associated with a fractured coronary stent has only rarely been described.

We report a rare case of a chronic peri-stent fibro-inflammatory fistulizing process associated with a fractured right coronary artery (RCA) stent presenting nearly seven years after PCI as a persistent draining xiphisternal sinus. This case highlights the diagnostic challenges posed by delayed peri-stent inflammatory disease, the broad differential diagnosis of persistent chest wall sinus following coronary intervention, and the pivotal role of multimodality imaging in defining the anatomical extent of disease and guiding successful surgical management.

## Case presentation

A 53-year-old man with a 5-year history of type 2 diabetes mellitus and ischemic heart disease underwent primary percutaneous coronary intervention (PCI) with right coronary artery (RCA) stenting for an acute inferior wall ST-elevation myocardial infarction in 2018. He remained clinically stable until 2024, when he began experiencing recurrent intermittent febrile episodes. During this period, he received multiple courses of empirical outpatient antibiotic therapy, including oral amoxicillin-clavulanate (625 mg) and clindamycin for 10 days, with only transient symptomatic improvement.

In June 2025, the patient developed a localized swelling over the xiphisternal region, which was managed at another hospital as a presumed sebaceous cyst/chest wall abscess by incision and drainage (Fig. 1).

**Fig. 1:**
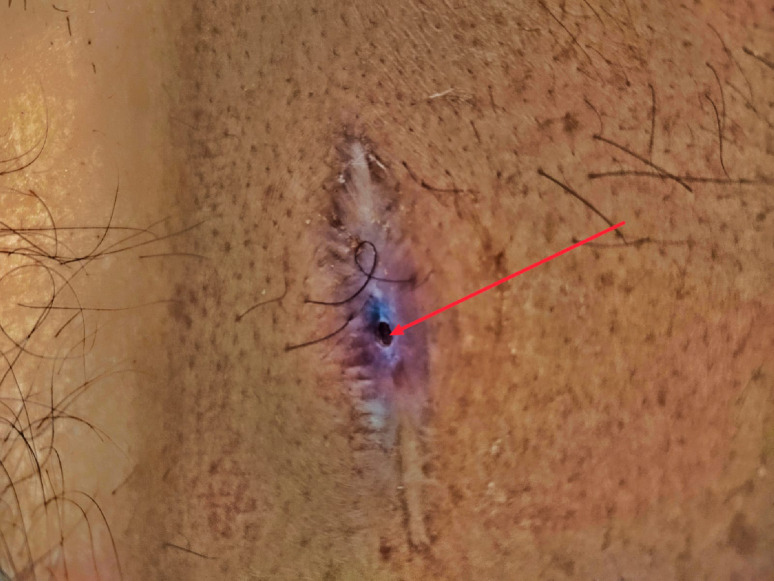
Clinical photograph of the xiphisternal cutaneous fistula. (Clinical photograph showing a small draining cutaneous fistulous opening in the xiphisternal/subcostal region (red arrow) with surrounding induration and post-debridement changes. The lesion was initially managed as a presumed sebaceous cyst/chest wall abscess; however, persistent drainage prompted further multimodality imaging, which demonstrated communication with an underlying peri-coronary fibro-inflammatory process. Abbreviation: RCA, right coronary artery)

Wound cultures obtained at that time demonstrated no microbial growth, likely reflecting prior prolonged antibiotic exposure. Despite the procedure, he continued to have intermittent low-grade fever and subsequently developed a persistent draining xiphisternal sinus over the following seven months.

Laboratory investigations demonstrated elevated inflammatory markers, with a C-reactive protein (CRP) level of 76 mg/L and an erythrocyte sedimentation rate (ESR) of 50 mm/hour at initial evaluation. Despite empirical antibiotic therapy, inflammatory markers remained elevated during follow-up (CRP 72 mg/L and ESR 65 mm/hour), consistent with an ongoing chronic inflammatory process. Routine hematological and biochemical investigations were otherwise unremarkable.

Transthoracic echocardiography (TTE) demonstrated mild left ventricular systolic dysfunction with a left ventricular ejection fraction (LVEF) of 45–50%, inferior wall regional wall motion abnormalities, grade II left ventricular diastolic dysfunction, and a small circumferential pericardial effusion. Transesophageal echocardiography (TEE) was not performed because there was no clinical or transthoracic echocardiographic evidence of infective endocarditis, and cross-sectional imaging adequately delineated the anatomical extent of the lesion.

Initial ultrasonography of the anterior chest wall demonstrated a well-defined irregular hypoechoic collection measuring 3.6 × 1.4 × 2.5 cm (approximately 7 mL) within the subcutaneous plane of the xiphisternal region without significant surrounding soft-tissue edema or definite intrathoracic extension.

Because of the persistent draining sinus and atypical clinical course, contrast-enhanced computed tomography (CT) of the chest was performed, demonstrating a hypodense inflammatory collection in the subxiphoid region with extension along the course of the RCA, raising suspicion for deeper peri-coronary pathology (Fig. 2). CT coronary angiography (CTCA) was performed instead of invasive coronary angiography to characterize the suspected peri-coronary pathology. Following CTCA, cardiac MRI confirmed non-viable myocardium in the RCA territory, supporting the decision to defer CABG and obviating the need for invasive coronary angiography. CTCA demonstrated a fractured and chronically occluded stent involving the proximal and mid RCA, surrounded by extensive peri-stent inflammatory soft tissue extending into the anterior pericardium, diaphragm, and xiphoid region. An associated anterior perihepatic collection measuring 2.16 × 3.30 cm and multiple enlarged anterior cardiophrenic lymph nodes measuring up to 9.5 × 11 mm were identified. Mild eccentric atherosclerotic calcification was present in the left anterior descending and left circumflex arteries, with an Agatston calcium score of 44.4 AU (Fig. 2). CTCA demonstrated distal collateral filling of the posterior descending artery from branches of the left circumflex artery.

**Fig. 2:**
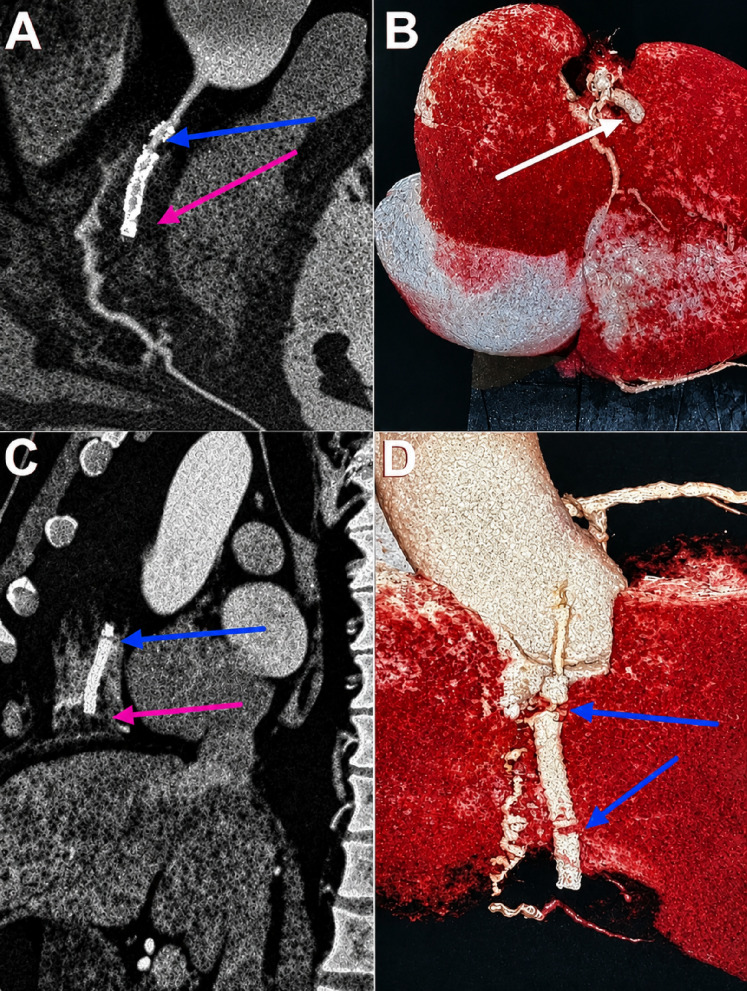
Contrast-enhanced CT chest and CT coronary angiography demonstrating a fractured right coronary artery (RCA) stent with chronic peri-stent fibro-inflammatory changes. (A) Curved multiplanar reformatted CT coronary angiography image demonstrating a fractured stent within the proximal-to-mid RCA (blue arrow) surrounded by peri-stent fibro-inflammatory soft tissue (pink arrow). (B) Three-dimensional volume-rendered CT coronary angiography demonstrating chronic total occlusion of the proximal RCA at the site of the fractured stent (white arrow). (C) Coronal contrast-enhanced CT image demonstrating peri-stent fibro-inflammatory soft tissue extending from the RCA along the anterior pericardium toward the xiphoid region (pink arrow). (D) Three-dimensional volume-rendered reconstruction demonstrating complete stent fracture (blue arrows) with distal collateral reconstitution of the RCA territory. CT, computed tomography; CTCA, computed tomography coronary angiography; RCA, right coronary artery

Cardiac magnetic resonance imaging (MRI) further characterized the lesion by demonstrating an enhancing periarterial soft-tissue lesion of intermediate T1- and T2-weighted signal intensity surrounding the right coronary artery (RCA), with contiguous extension from the right atrioventricular groove into the cardiophrenic region involving the adjacent pericardium, mediastinal pleura, and anterior diaphragm. Small fluid collections were identified within the cardiophrenic and epiphrenic regions. A fistulous tract extending from the inflammatory collection to the overlying xiphisternal skin confirmed cutaneous fistulization. The overall craniocaudal extent of the fibro-inflammatory process measured approximately 12 cm (Fig. 3). Late gadolinium enhancement demonstrated non-viable myocardium in the RCA territory, consistent with chronic infarction, supporting the decision to defer coronary artery bypass grafting (CABG). Overall, cardiac MRI accurately delineated the anatomical extent of the peri-stent fibro-inflammatory process and played a pivotal role in surgical planning.

**Fig. 3:**
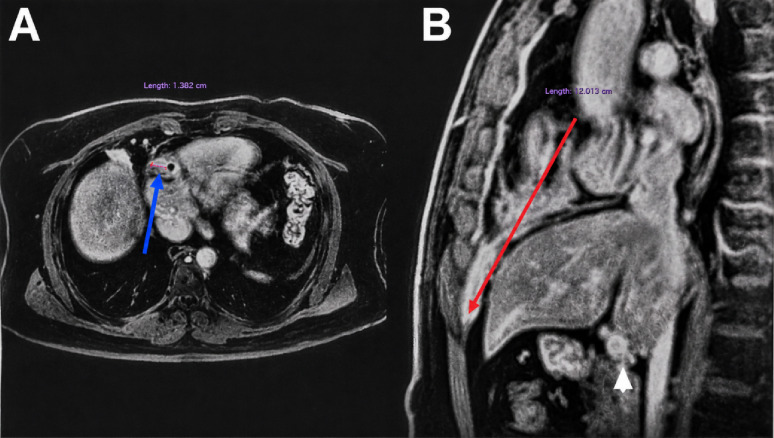
Cardiac magnetic resonance imaging demonstrating chronic peri-stent fibro-inflammatory extension and cutaneous fistulization. (A) Axial post-contrast cardiac magnetic resonance image demonstrating enhancing periarterial fibro-inflammatory tissue surrounding the RCA within the right atrioventricular groove (blue arrow). (B) Coronal post-contrast image demonstrating contiguous extension of the fibro-inflammatory process involving the pericardium, mediastinal pleura, and anterior diaphragm (red arrow), with an associated epiphrenic fluid collection and a fistulous tract extending toward the xiphisternal skin surface (white arrow). Abbreviation: MRI, magnetic resonance imaging; RCA, right coronary artery

Overall, multimodality imaging demonstrated a chronic peri-stent fibro-inflammatory fistulizing process centered around the fractured RCA stent with extensive mediastinal and transdiaphragmatic extension.

The patient underwent off-pump median sternotomy. Dense pericardial adhesions were carefully released using a harmonic scalpel. Extensive chronic fibro-inflammatory tissue was identified surrounding the proximal and mid RCA along the course of the fractured stent. A localized peri-stent inflammatory cavity communicating with the chronic xiphisternal sinus tract was identified (Fig. 4).

**Fig. 4:**
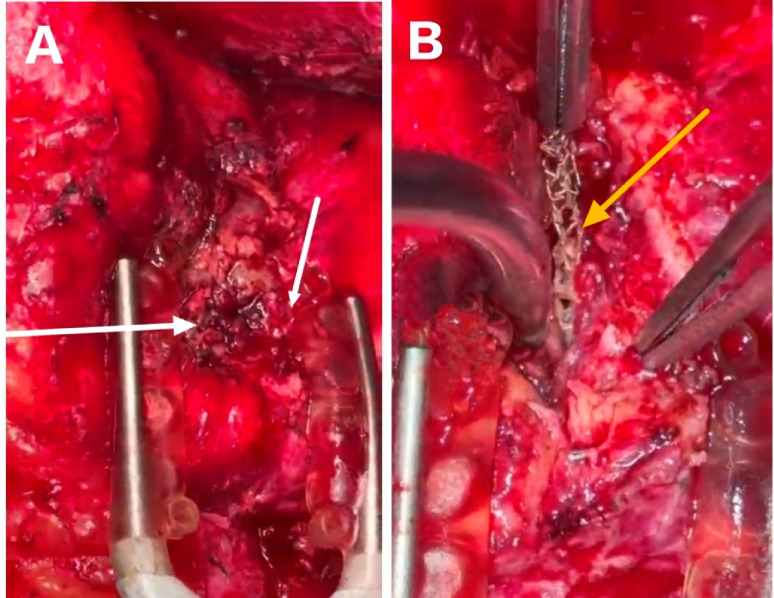
Intraoperative findings during surgical exploration. (A) Intraoperative photograph demonstrating a chronic fibro-inflammatory cavity with dense fibrotic tissue surrounding the course of the right coronary artery (white arrows). (B) Surgical extraction of the fractured RCA stent (yellow arrow) together with the surrounding chronic fibro-inflammatory tissue following meticulous debridement. RCA, right coronary artery

The involved RCA segment was meticulously dissected, and the fractured RCA stent, together with the chronic sinus tract and surrounding fibro-inflammatory tissue, was completely excised en bloc. Marsupialization of the chronic peri-stent inflammatory cavity was performed following complete excision of the fractured stent and surrounding fibro-inflammatory tissue. CABG was deferred because of chronic total occlusion of the RCA, non-viable myocardium demonstrated on cardiac MRI, and satisfactory distal collateral circulation. The patient received perioperative intravenous piperacillin-tazobactam and linezolid, followed by oral cefixime-clavulanate and levofloxacin postoperatively.

Wound swab cultures, excised tissue cultures, and intraoperative microbiological studies demonstrated no microbial growth. GeneXpert testing for *Mycobacterium tuberculosis* was negative. Specific microbiological investigations for nontuberculous mycobacteria, including prolonged mycobacterial culture, were not performed. Although prolonged antecedent antibiotic therapy may have contributed to sterile cultures, a chronic sterile fibro-inflammatory process could not be completely excluded.

The postoperative course was uneventful. The patient received completion of empiric postoperative antimicrobial therapy. and was discharged on guideline-directed secondary prevention comprising single antiplatelet therapy, high-intensity statin therapy, and a beta-blocker. Serial follow-up at 15 days, 1 month, 3 months, and 6 months demonstrated complete healing of the xiphisternal wound without recurrent drainage, fever, or fistula formation. Follow-up transthoracic echocardiography showed no pericardial collection or valvular vegetations, a left ventricular ejection fraction of 50%, and persistent inferior wall hypokinesia.

The chronological sequence of clinical events is summarized in (Table 1).

**Table 1:** Chronological sequence of clinical presentation, multimodality imaging findings, surgical management, and follow-up in a patient with a fractured right coronary artery stent associated with a chronic peri-stent fibro-inflammatory process and xiphisternal cutaneous fistulization

Timeline	Clinical event	Findings/outcome
2018	Percutaneous coronary intervention (PCI) with right coronary artery (RCA) stenting	RCA stent placement for ischemic heart disease
2024	Onset of recurrent intermittent febrile episodes	Persistent low-grade febrile illness with unclear etiology
June 2025	Development of localized xiphisternal swelling	Initially presumed to represent sebaceous cyst/chest wall abscess
June 2025	Surgical incision and drainage performed	Persistent postoperative serosanguineous discharge; fluid cultures negative following prolonged antibiotic therapy
Following 7 months	Persistent draining xiphisternal sinus	Associated intermittent low-grade fever and non-healing cutaneous fistula
Initial imaging evaluation	Ultrasound (USG) anterior chest wall	Irregular hypoechoic subcutaneous collection measuring 3.6 × 1.4 × 2.5 cm (~ 7 cc) without definite intrathoracic extension
Further cross-sectional imaging	Contrast-enhanced CT (CECT) chest	Subxiphoid/perixiphoid inflammatory collection extending along RCA course
Advanced coronary imaging	CT coronary angiography (CTCA)	Fractured and completely occluded proximal-mid RCA stent with peri-stent inflammatory soft tissue extending into anterior pericardium and subdiaphragmatic region; associated perihepatic collection
Further tissue characterization	Cardiac magnetic resonance imaging (MRI)	Extensive periarterial inflammatory soft tissue involving pericardium, mediastinal pleura, and diaphragm with cutaneous fistulous communication; total inflammatory extent ~ 12 cm
Final diagnosis	Multimodality imaging diagnosis	Chronic peri-stent inflammatory/infective process associated with fractured RCA stent and transdiaphragmatic cutaneous fistulization
Surgical management	Operative exploration and debridement	Complete excision of chronic fistulous tract; removal of fractured RCA stent with debridement of dense fibro-inflammatory tissue; marsupialization of involved RCA segment
Postoperative course	Recovery and follow-up	Uneventful postoperative recovery with complete healing of xiphisternal wound and no recurrent drainage or fever

RCA, right coronary artery

## Discussion

Coronary stent-related inflammatory complications are exceptionally uncommon but potentially life-threatening sequelae of percutaneous coronary intervention (PCI), with only a limited number of cases reported in the literature [1–3]. Most published cases occur within days to weeks after PCI and are attributed to procedural contamination, transient bacteremia, or hematogenous seeding of the implanted stent [1–3]. In contrast, delayed presentations occurring approximately 6–7 years after PCI are exceedingly rare and present considerable diagnostic challenges because of their indolent clinical course and nonspecific manifestations [6, 7].

The present case is unique because of the coexistence of a complete fracture of an everolimus-eluting Xience Alpine right coronary artery (RCA) stent and an extensive chronic peri-stent fibro-inflammatory process with contiguous extension into the pericardium, mediastinum, diaphragm, and subdiaphragmatic space, ultimately resulting in a chronic cutaneous xiphisternal fistula. Such transcompartmental inflammatory extension has rarely been described and may initially mimic superficial chest wall conditions such as an infected sebaceous cyst, chronic chest wall abscess, or osteomyelitis, thereby delaying recognition of the underlying cardiovascular pathology.

The precise pathogenesis remains uncertain and is likely multifactorial. Histopathological examination demonstrated marked disruption of the arterial media at the stent strut sites with dense inflammatory infiltrates composed predominantly of macrophages, lymphocytes, and multinucleated foreign-body giant cells. The surrounding tissue exhibited severe chronic active inflammation, granulation tissue, focal microabscess formation, fibrin-platelet deposition, and necrotic debris, while Gram staining demonstrated no microorganisms. These findings support a chronic fibro-inflammatory response centered on the fractured stent but do not establish a definitive infectious etiology. Mechanical disruption associated with complete stent fracture may have resulted in persistent endothelial injury, altered local hemodynamics, and continuous tissue irritation, thereby facilitating progressive peri-stent inflammation and extension into adjacent mediastinal structures. An unrecognized chronic micro-perforation or a contained peri-coronary pseudoaneurysm secondary to mechanical disruption cannot be excluded and may represent an additional mechanism for progressive extra-coronary inflammatory extension, although neither was definitively demonstrated by imaging or surgery. Previous studies have demonstrated that coronary stent fracture is associated with chronic vascular injury, restenosis, thrombosis, and persistent inflammatory responses, although progression to extensive fistulizing mediastinal disease remains exceptionally uncommon [8, 9].

Despite the absence of microbiological confirmation, a culture-negative infectious process cannot be completely excluded. Three sets of blood cultures, wound cultures, excised tissue cultures, fungal cultures, and cultures for *Actinomyces* and *Nocardia* were all negative. Gram staining and acid-fast bacilli staining demonstrated no microorganisms, and GeneXpert testing for *Mycobacterium tuberculosis* was negative. However, the patient had received prolonged empirical antibiotic therapy before referral, followed by broad-spectrum perioperative antimicrobial therapy, which may have substantially reduced microbiological yield. Furthermore, microorganisms embedded within biofilms on implanted cardiovascular devices may evade conventional culture techniques despite persistent chronic inflammation because of reduced metabolic activity and limited antimicrobial penetration [10, 11]. Conversely, the prominent foreign-body giant-cell reaction identified histologically raises the possibility of a sterile inflammatory response or delayed hypersensitivity reaction to the fractured everolimus-eluting stent, a mechanism previously described with drug-eluting coronary stents [12].

The differential diagnosis included chronic bacterial mediastinitis, tuberculous sinus tract, nontuberculous mycobacterial infection, fungal infection, actinomycosis, nocardiosis, chronic osteomyelitis, granulomatous inflammatory disorders, and sterile foreign-body reaction. Tuberculosis was considered unlikely because GeneXpert and acid-fast bacilli staining were negative. However, prolonged cultures specific for nontuberculous mycobacteria were not performed; therefore, this possibility cannot be completely excluded. Nevertheless, the characteristic peri-coronary distribution centered on the fractured stent, together with the operative findings, histopathological features, elevated inflammatory markers, and complete clinical resolution after surgical excision without recurrence during six months of follow-up, strongly supports a stent-associated chronic fibro-inflammatory process as the principal pathological substrate.

Multimodality imaging played a pivotal role in establishing the diagnosis and planning surgical management. Initial ultrasonography identified only a superficial subcutaneous collection and failed to demonstrate the full extent of disease. Contrast-enhanced computed tomography suggested deeper peri-coronary involvement, while CT coronary angiography accurately demonstrated complete stent fracture, chronic total occlusion of the RCA, and extensive peri-stent fibro-inflammatory soft tissue. CTCA is an established non-invasive modality for evaluating coronary stent integrity and associated complications in selected patients [5, 13]. CTCA also demonstrated preserved distal perfusion through well-developed collateral vessels arising from the left circumflex artery supplying the distal posterior descending artery. Cardiac magnetic resonance imaging provided superior soft-tissue characterization by delineating involvement of the pericardium, mediastinal pleura, diaphragm, and the cutaneous fistulous tract while simultaneously demonstrating non-viable myocardium within the RCA territory [4]. Collectively, these findings explained why invasive coronary angiography was unlikely to alter management and supported proceeding directly to definitive surgical treatment.

The surgical strategy was guided by the anatomical information obtained from multimodality imaging. Off-pump median sternotomy allowed complete excision of the fractured stent together with the chronic fibro-inflammatory tract and surrounding diseased tissue while preserving healthy myocardium. Marsupialization of the inflammatory cavity facilitated adequate drainage and debridement. Coronary artery bypass grafting was intentionally deferred because cardiac magnetic resonance imaging demonstrated non-viable myocardium within the RCA territory, and CTCA confirmed satisfactory collateral perfusion of the distal posterior descending artery from the left circumflex artery, indicating that surgical revascularization was unlikely to provide additional functional benefit.

Although transesophageal echocardiography (TEE) is valuable for evaluating infective endocarditis, peri-annular extension, and intracardiac complications, it was not performed in the present case because transthoracic echocardiography demonstrated no valvular vegetations or features suggestive of infective endocarditis, and multimodality cross-sectional imaging adequately characterized the extra-cardiac disease and provided sufficient information for surgical planning. Similarly, endoscopic ultrasound has been described as a useful adjunct for evaluating selected mediastinal collections adjacent to the esophagus. However, because the inflammatory process in this patient was predominantly anterior, peri-coronary, and transdiaphragmatic, CTCA and cardiac magnetic resonance imaging comprehensively delineated the disease extent, making additional endoscopic imaging unlikely to influence management.

This case highlights several important clinical lessons. Persistent or recurrent anterior chest wall sinus formation in patients with previous coronary intervention should prompt consideration of underlying mediastinal or peri-coronary pathology rather than isolated superficial soft-tissue infection. Complete coronary stent fracture should be recognized as a potential contributor to chronic peri-stent inflammatory disease, particularly when accompanied by persistent inflammatory markers and atypical imaging findings. Multimodality imaging integrating CT, CT coronary angiography, and cardiac magnetic resonance imaging is invaluable for defining disease extent, assessing myocardial viability, identifying fistulous communication, and guiding individualized surgical management. Early multidisciplinary collaboration among cardiologists, cardiovascular radiologists, cardiothoracic surgeons, infectious disease specialists, microbiologists, and pathologists is essential for optimizing outcomes in these complex patients.

This report has several limitations. Despite extensive microbiological investigation, including repeated blood cultures, wound and tissue cultures, fungal cultures, cultures for *Actinomyces* and *Nocardia*, acid-fast bacilli staining, and GeneXpert testing, no causative microorganism was identified. Specific cultures for nontuberculous mycobacteria and advanced molecular diagnostic techniques, such as broad-range 16 S ribosomal RNA sequencing, were not performed and might have improved microbiological characterization. Histopathology confirmed chronic active fibro-inflammatory changes with foreign-body giant-cell reaction but could not establish a definitive infectious etiology. Invasive coronary angiography was not performed because CT coronary angiography and cardiac magnetic resonance imaging provided sufficient anatomical and functional information to guide management. Finally, although serial clinical and echocardiographic follow-up at 15 days, 1 month, 3 months, and 6 months demonstrated complete wound healing without recurrent fistula, fever, or inflammatory manifestations, longer-term follow-up would further strengthen assessment of durability.

To our knowledge, chronic xiphisternal cutaneous fistulization associated with a fractured everolimus-eluting RCA stent and extensive transdiaphragmatic peri-stent fibro-inflammatory extension remains an exceptionally rare presentation. This case underscores the importance of maintaining a high index of suspicion for delayed stent-related inflammatory complications, highlights the indispensable role of multimodality imaging in diagnosis and operative planning, and demonstrates that timely multidisciplinary surgical management can achieve excellent clinical outcomes despite the absence of definitive microbiological confirmation.

## Conclusions

This case describes an exceptionally rare chronic peri-stent fibro-inflammatory fistulizing process associated with a fractured right coronary artery stent presenting as a persistent xiphisternal draining sinus approximately 6–7 years after PCI. It highlights the potential for extensive contiguous mediastinal and transdiaphragmatic extension, which may mimic superficial chest wall pathology and delay diagnosis. Multimodality imaging with CT coronary angiography and cardiac magnetic resonance imaging was pivotal in defining the anatomical extent of disease, assessing myocardial viability, and guiding surgical management. In patients with a history of PCI who present with persistent chest wall sinus formation or unexplained recurrent fever, clinicians should consider the possibility of underlying peri-coronary pathology, even when conventional microbiological investigations are negative.

## Learning points

- Delayed peri-stent fibro-inflammatory complications can rarely occur several years after PCI and may present with atypical clinical manifestations.
- Coronary stent fracture should be considered a potential contributor to chronic peri-stent inflammatory changes when supported by imaging and operative findings.
- Persistent anterior chest wall sinus or recurrent unexplained fever in patients with previous coronary intervention should prompt evaluation for deeper mediastinal or peri-coronary pathology.
- Multimodality imaging, particularly CT coronary angiography and cardiac magnetic resonance imaging, is valuable for defining disease extent, assessing myocardial viability, and planning surgical management.
- Negative microbiological cultures do not completely exclude an underlying inflammatory or culture-negative infectious process, particularly after prolonged antibiotic exposure.
- Early multidisciplinary collaboration involving cardiology, cardiovascular imaging, infectious disease specialists, pathology, and cardiothoracic surgery is essential in managing complex peri-stent inflammatory lesions.

## Data Availability

No datasets were generated or analysed during the current study.

## Abbreviations

PCI: Percutaneous coronary intervention
RCA: Right coronary artery
CT: Computed tomography
CECT: Contrast-enhanced computed tomography
CTCA: Computed tomography coronary angiography
MRI: Magnetic resonance imaging
USG: Ultrasonography
LV: Left ventricle
LVEF: Left ventricular ejection fraction
AV: Atrioventricular
cm: Centimeter
CRP: C-reactive protein
ESR: Erythrocyte sedimentation rate
CABG: Coronary artery bypass grafting
